# Incident and Adverse-Event Reporting in Undergraduate Dental Education: A Scoping Review of Educational Evidence, Curricular Integration, Perceptions, and Barriers

**DOI:** 10.3390/dj14100655

**Published:** 2026-10-09

**Authors:** Vivek Padmanabhan, Megan Brown, Md Sofiqul Islam, Nada Tawfig Hashim, Muhammed Mustahsen Rahman

**Affiliations:** 1 RAK College of Dental Sciences, RAK Medical & Health Sciences University, Ras-AL Khaimah 12973, United Arab Emirates; sofiqul.islam@rakmhsu.ac.ae (M.S.I.);; 2 School of Medicine, Newcastle University, Newcastle upon Tyne NE2 4HH, UK

**Keywords:** patient safety, medical errors, patient harm, risk management, students, dental, education, dental, clinical competence

## Abstract

Incident and adverse-event reporting is an important component of patient safety and provides opportunities for risk reduction and organizational learning. In undergraduate dental education programmes, students deliver patient care as they develop clinical competence; however, the extent to which incident reporting is taught, practiced, and integrated into clinical education or the dental curriculum remains unclear. This scoping review mapped the available evidence on the reporting of incidents and adverse events in undergraduate dental education. The review was undertaken in accordance with the framework of Arksey and O’Malley, JBI guidance, and PRISMA-ScR. Scopus, PubMed, Web of Science, and Google Scholar were searched for English-language studies published from 2015 to 2026 that addressed incident or adverse-event reporting in undergraduate dental education programmes, and data were collated and synthesized descriptively and thematically. Thirteen studies met the inclusion criteria. Students’ knowledge, confidence, and preparedness for incident reporting varied widely, and reporting was more often taught within general patient-safety education than as a distinct subject. Underreporting was common, particularly for incidents perceived as minor. Recurring barriers included fear of adverse consequences, limited support within the clinical hierarchy, and uncertainty about how to report. Few studies described any formal assessment of reporting competence, whereas studies in which adverse events were reviewed and fed back to students pointed to clear educational value. Undergraduate dental education addresses incident and adverse-event reporting inconsistently; clear reporting pathways, supportive supervision, constructive feedback, and a non-punitive clinical environment may strengthen reporting competence and patient-safety learning.

## 1. Introduction

Incident reporting underpins patient safety by identifying risks, clarifying the circumstances in which adverse events arise, and generating learning at the organizational level [1]. Since To Err Is Human appeared in 2000, health systems have placed growing weight on systematic identification and reporting of near misses and adverse events as a route to reducing preventable harm [2]. That emphasis has extended into the education of health professionals, with patient-safety concepts and competencies increasingly built into undergraduate curricula [3].

In the field of patient safety, incidents include events or circumstances that could have resulted, or did result, in unnecessary harm to a patient, while adverse events refer specifically to incidents that resulted in harm to patients [4]. Near misses are incidents that do not reach the patient, whereas clinical errors involve failures in the execution or planning of care and may or may not result in harm [4]. Reporting incidents refers to formal communication and documentation of such events through established mechanisms within the organization. This is distinct from incident or adverse event disclosure, which includes communication of the error or harm that occurred to the affected patient and family, it also involves providing support to the patient to overcome the situation [1,4].

Incident and adverse-event reporting is undoubtedly critical to undergraduate dental education programmes as students provide direct patient care early in their educational career while progressively developing their clinical knowledge, technical skills, and professional judgement. Dental clinical training involves a range of diagnostic, preventive and therapeutic procedures in which errors, near misses, and adverse events may occur, creating opportunities for students to identify patient safety concerns and learn appropriate responses. However, meaningful learning from such events requires more than clinical exposure alone; students need to understand what essentially is included as an incident which can be reported, how, when and to whom it should be reported, and the institutional mechanism through which management, feedback and subsequent learning occur. Integrating these competencies into undergraduate training programmes may therefore help students develop an understanding of incident reporting as both a professional responsibility and a mechanism for organizational learning [5,6].

The clinical environment of undergraduate dental training gives rise to a characteristic range of incidents. Analyses of national incident databases and malpractice series indicate that the events encountered most frequently in dental care include soft-tissue injury, hard-tissue damage to adjacent or opposing teeth, aspiration or ingestion of instruments and materials, complications of local anesthesia such as prolonged paraesthesia and nerve injury, endodontic mishaps including instrument separation and extrusion of irrigant beyond the apex, post-operative infection and hemorrhage, medication and prescribing errors, and failures of record keeping or documentation [7,8]. A smaller number of events are classified as never or serious events, of which wrong-tooth and wrong-site extraction is the most frequently reported in dentistry and has prompted the introduction of pre-operative checklists and site-marking protocols in both hospital and teaching settings [9,10]. Occupational incidents affecting the student are also common: needlestick and sharps injuries have an estimated pooled prevalence of approximately 44% among dental students worldwide and are consistently under-reported, with many students unaware that such exposures should be reported at all [11]. Students may additionally be involved in the recognition and management of medical emergencies occurring in the dental chair, and in breaches of infection-control procedure. Many of these events are minor or result in no harm to the patient, and it is precisely this category of near miss and low-harm incident that is most susceptible to under-reporting and most valuable as a source of learning.

Existing literature suggests that patient safety is increasingly recognized within undergraduate dental education, with studies examining students’ knowledge, attitudes, perceptions, and confidence, as well as institutional responses to serious patient-safety events and the integration of patient-safety content within dental curricula [12,13,14]. However, evidence concerning patient safety education more broadly should be distinguished from evidence specifically addressing incidents and adverse-event reporting. General patient safety competencies, including communication, teamwork, infection prevention, and safety awareness, provide an important context for safe clinical practice but do not necessarily indicate that students are taught how to recognize reportable incidents, use institutional reporting systems, or participate in subsequent reporting and learning processes [14]. Although incident-reporting mechanisms have been described within undergraduate dental education, their application may extend beyond reporting of patient adverse events to areas such as the assessment of professional and unprofessional behaviours [15].

Patient safety has drawn increasing attention in dental education, yet the evidence dealing specifically with incident and adverse-event reporting among undergraduate students is limited and heterogeneous. Available studies have examined various aspects of patient-safety education and reporting, but the extent to which reporting is explicitly taught, practiced, and assessed within undergraduate dental education remains unclear. Mapping this evidence is important for identifying what is currently known and where educational practices remain insufficiently described or evaluated. Therefore, this scoping review aimed to map the available evidence on incident and adverse-event reporting in undergraduate dental education, with particular emphasis on how reporting is taught, practiced, and assessed. It also sought to examine students’ knowledge, perceptions, confidence, and reported behaviours related to incident reporting, identify curricular and institutional factors influencing reporting, and highlight gaps in the existing educational evidence.

## 2. Materials and Methods

### 2.1. Study Design and Framework

A scoping review was undertaken to map the available evidence on incident and adverse-event reporting in undergraduate dental education. The review methodology was in accordance with framework proposed by Arksey and O’Malley [16] and supplemented by the Joanna Briggs Institute (JBI) guidance for scoping reviews [17]. The preferred Reporting Items for Systematic Reviews and Meta-Analyses extension for Scoping Reviews (PRISMA-ScR) framework was used to report the review to promote transparent and reproducible reporting of the review process [18]. An a priori review protocol was developed as part of an academic medical education programme and was reviewed by academic supervisors before the search was conducted. The protocol was not prospectively registered in a public repository because PROSPERO, the principal international register for review protocols, does not accept registrations of scoping reviews; deposition in a general-purpose repository such as the Open Science Framework was not undertaken at the time. This is acknowledged as a limitation of the review. The completed PRISMA-ScR checklist is provided as Supplementary File S1.

### 2.2. Research Question

The following primary question guided the review: What evidence exists regarding how incident and adverse-event reporting is taught, practiced, and assessed in undergraduate dental education? Secondary areas of enquiry included students’ knowledge, perceptions, confidence, and reported behaviours related to incident reporting; curricular and institutional factors influencing reporting; and gaps in the available educational evidence.

### 2.3. Eligibility Criteria

The Population–Concept–Context (PCC) framework recommended by the Joanna Briggs Institute for scoping reviews was used to define the eligibility criteria for the review [17]. The target population comprised undergraduate dental students enrolled in dental programmes. Studies involving former dental students were also eligible when they specifically and retrospectively examined incident or adverse-event reporting experiences during undergraduate clinical training. Studies including mixed participant groups, such as undergraduate students, interns, postgraduate trainees, faculty, or other dental personnel, were eligible when undergraduate dental students formed part of the study population and the study contained findings directly relevant to incident or adverse-event reporting within the educational context.

The primary concept of interest was education related to incidents and adverse-event reporting. Eligible studies were required to address at least one reporting-specific component, including recognition of reportable incidents or near misses; knowledge or awareness of reporting procedures or systems; education or training related to reporting; use of institutional reporting systems; confidence, willingness, or intention to report; self-reported reporting experience or behaviour; barriers or facilitators to reporting; feedback or learning following reporting; or assessment of reporting-related knowledge or competence. Studies addressing broader patient safety knowledge, attitudes, culture, communication, teamwork, or clinical errors were included only when they contained an identifiable component directly relevant to incident or adverse-event reporting. Studies addressing occupational incidents affecting students during clinical training, such as sharps and percutaneous injuries, were also eligible where reporting behaviour, reporting systems, or barriers to reporting were examined, since these are managed through the same institutional reporting mechanisms and reporting culture as patient-safety incidents.

The context included educational, clinical, and simulation-based settings within undergraduate dental education. Primary quantitative, qualitative, mixed-methods, quality-improvement, and educational studies were eligible for inclusion. Studies were excluded if they focused exclusively on postgraduate education or practicing dental professionals; addressed patient safety without an identifiable incident- or adverse-event-reporting component; focused solely on error disclosure or speaking up without relevance to formal reporting; or were reviews, editorials, commentaries, conference abstracts, case reports, or other non-primary publications. English-language studies published between 2015 and 2026 were considered.

Several of these decisions warrant explanation. The search was restricted to studies published from 2015 onward in order to capture literature reflecting the period following widespread adoption of formal patient-safety curricula in health-professions education, by which time institutional incident-reporting systems had become a routine feature of the clinical teaching environment. Studies were limited to those published in English because translation resources were not available to the review team; this restriction is acknowledged as a limitation. Mixed participant groups were eligible only where undergraduate dental students formed part of the study population and the reporting-related findings were attributable to the educational context, so that conclusions about students were not drawn from data generated predominantly by qualified practitioners. Studies of former dental students were eligible only where the reported experiences related specifically and retrospectively to the undergraduate training period. Occupational incidents such as sharps and percutaneous injuries were included because they are managed through the same institutional reporting mechanisms and are governed by the same reporting culture as patient-safety incidents, and therefore inform the same educational question, although findings concerning harm sustained by the student may not generalize fully to harm sustained by the patient. Reviews, editorials, commentaries, case reports, and other non-primary publications were excluded because the review was designed to map primary evidence.

### 2.4. Search Strategy and Information Sources

An extensive literature search was undertaken across PubMed, Scopus, Web of Science, and Google Scholar on 20 May 2026, for studies published between 2015 and 2026. Search terms and controlled vocabulary relating to near misses, incidents, adverse events, patient safety, dental students, and undergraduate dental education programmes were combined using Boolean operators and adapted for each database. Google Scholar results were capped at the first 100 records ranked by relevance; these were reviewed against the eligibility criteria on the basis of title and displayed snippet, and 27 potentially relevant records were exported to EndNote for formal screening alongside the database records. This preliminary relevance filtering is acknowledged as a deviation from full screening of all retrieved records. Reference lists of the included studies were screened manually, and forward citation searching was undertaken using Google Scholar to identify additional eligible studies. The search strategy employed for each database is provided in Supplementary Table S1.

### 2.5. Study Selection Process

All identified records were exported to EndNote for initial deduplication and subsequently imported into Rayyan, where additional duplicate records were identified and removed before screening. Two reviewers (V.P. and M.S.I.) jointly screened an initial set of 30 records, selected in order of publication year, to calibrate the application of the eligibility criteria. Following this calibration exercise, the remaining titles and abstracts were independently screened by both reviewers. The full texts of potentially eligible studies were subsequently assessed independently by both reviewers. Disagreements were resolved through discussion, with a third reviewer (N.T.) available for adjudication when required. During full-text assessment, particular attention was given to confirming the presence of an identifiable component related to incident or adverse-event reporting rather than patient safety more broadly. The study-selection process was documented using a PRISMA-ScR flow diagram [18].

### 2.6. Data Extraction

Data were extracted using a structured Microsoft Excel form developed for the review and pilot-tested on a subset of included studies. The extracted variables included author, year of publication, country, study design, participant characteristics, educational or clinical context, reporting-related educational approaches, reporting systems or processes, assessment methods, and key findings related to knowledge, attitudes, behaviours, barriers, and facilitators of incident or adverse-event reporting. The specific relevance of each study to incident or adverse-event reporting was also recorded. Data extraction was performed by one reviewer (VP) and independently verified for accuracy and completeness by a second reviewer (SI). Any discrepancies were resolved through discussion.

### 2.7. Critical Appraisal

Formal assessment of methodological quality or risk of bias was not performed because the review aimed to map the nature and extent of the available evidence rather than evaluate intervention effectiveness or certainty of evidence. This is consistent with established methodological guidance for scoping reviews [16,17].

### 2.8. Data Analysis and Synthesis

Extracted data were analyzed descriptively and narratively. Study characteristics, including publication year, country, study design, participant characteristics, and educational or clinical context, was summarized descriptively. Reporting-specific findings were then coded and grouped according to recurring concepts related to incident and adverse-event reporting. These included recognition and education concerning reportable events, reporting behaviours and underreporting, reporting systems and learning from reported events, and barriers and facilitators to reporting. Given the heterogeneity of study designs, settings, and outcomes, findings were synthesized narratively to identify patterns, gaps, and areas of convergence across the included studies.

### 2.9. Ethical Considerations

As this review synthesized data from publicly available published literature and as no human participants or identifiable individual-level data were involved, ethical approval was not required.

## 3. Results

### 3.1. Study Selection Results

**Figure 1:**
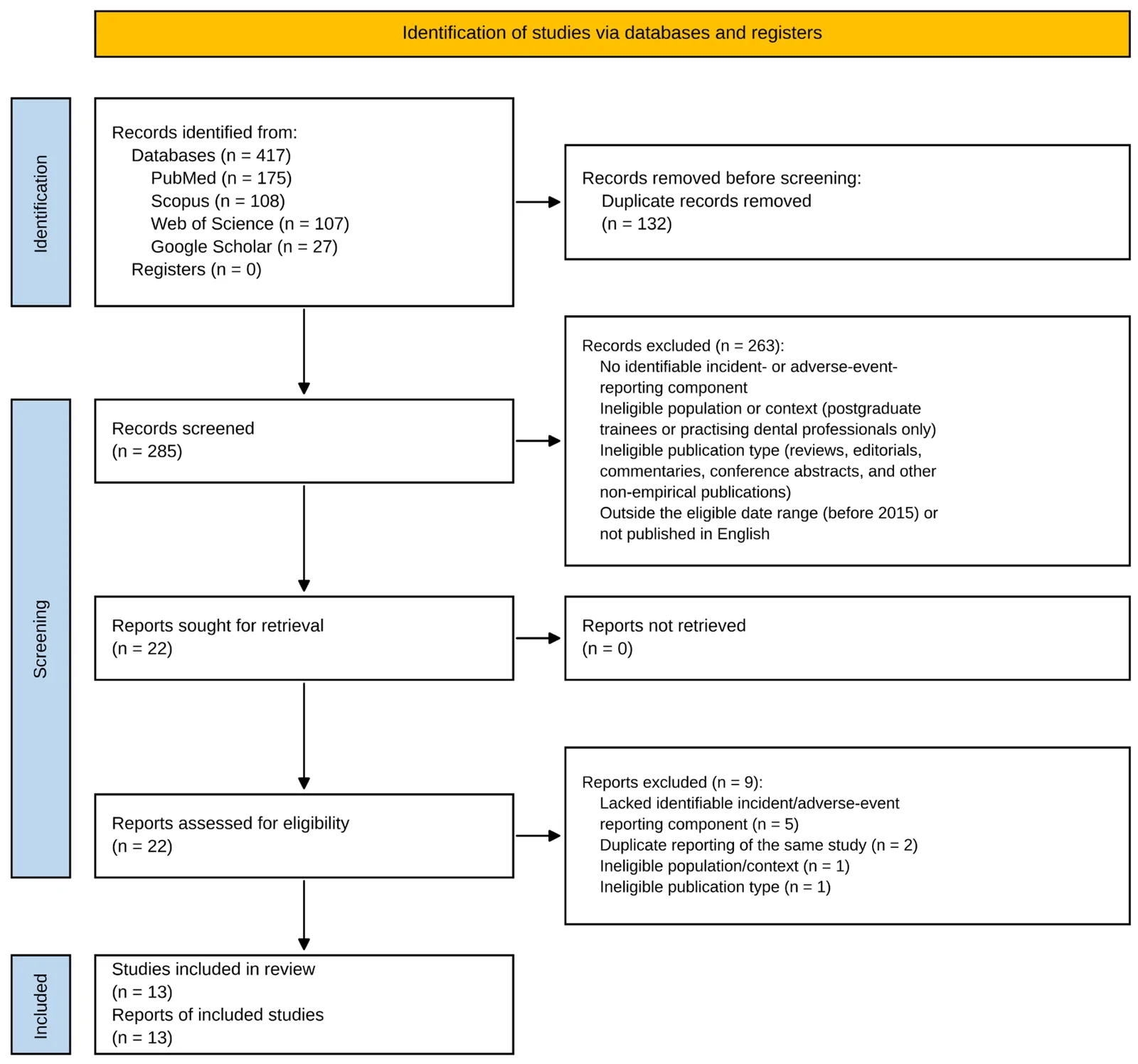
PRISMA-ScR flow diagram illustrating the identification of records through database searching, and the screening, eligibility assessment, and inclusion of studies in the scoping review. Records were excluded at title and abstract screening against the eligibility criteria shown. Counts by individual reason are not reported at this stage because records frequently met more than one exclusion criterion; numerical reasons are reported for full-text exclusions.

Database searching returned 417 records (PubMed, *n* = 175; Scopus, *n* = 108; Web of Science, *n* = 107; Google Scholar, *n* = 27), of which 132 duplicates were removed. Of the 285 records taken to title and abstract screening, 263 did not meet the eligibility criteria and were excluded for the following reasons: no identifiable component related to incident or adverse-event reporting; ineligible population or context (postgraduate trainees or practicing dental professionals only, with no undergraduate component); ineligible publication type (reviews, editorials, commentaries, conference abstracts, or non-empirical publications); or records falling outside the eligible date range (pre-2015) or published in a non-English language. Twenty-two full texts were then assessed for eligibility, of which nine were excluded: five had no identifiable component related to incident or adverse-event reporting, two were duplicate reports of the same study, one fell outside the eligible population or context, and one was an ineligible publication type. We screened the reference lists of the included studies and conducted forward citation searches in Google Scholar; neither identified additional studies meeting the eligibility criteria. These supplementary searches are not represented in Figure 1, which reflects identification via database searching. Thirteen studies were included. Figure 1 presents the selection process as a PRISMA-ScR flow diagram [18].

### 3.2. Characteristics of Included Studies

The thirteen included studies were published between 2017 and 2025 and came from eleven countries: the United Kingdom, the United States, Saudi Arabia, Pakistan, the United Arab Emirates, Türkiye, Iran, Greece, Mexico, Colombia, and Jordan [13,14,19,20,21,22,23,24,25,26,27,28,29]. Cross-sectional and other observational designs predominated, but the evidence base also contained curriculum-based evaluations, retrospective analyses of adverse events, one educational intervention, and one institutional quality-improvement study [13,14,19,20,21,22,23,24,25,26,27,28,29]. Study populations predominantly comprised undergraduate dental students, particularly those in their clinical years. Some studies included mixed participant groups, including interns, faculty, dental-assisting students, or other clinical personnel, from which findings relevant to undergraduate dental education could be identified [14,19,27,28]. One study retrospectively examined dentists’ experiences of adverse events occurring during their undergraduate clinical training [22]. The studies varied considerably in their approach to incident and adverse-event reporting. These included assessment of students’ knowledge and confidence in reporting, analysis of adverse events encountered or reported during clinical training, reporting of sharps and percutaneous injuries, evaluation of reporting barriers and underreporting, curriculum-based patient-safety education, and institutional reporting systems with mechanisms for review, feedback, and learning [13,14,19,20,21,22,23,24,25,26,27,28,29]. The characteristics of the included studies and their specific relevance to incident or adverse-event reporting are summarized in Table 1.

### 3.3. Reporting-Specific Evidence Synthesis

#### 3.3.1. Recognition of Reportable Events and Reporting-Related Education

The included studies showed considerable variation in how undergraduate dental students were prepared to recognize and respond to reportable patient-safety events. Reporting-related education was commonly embedded within broader patient-safety teaching rather than delivered as a distinct curricular component. Dargue et al. examined undergraduate dental schools’ experience of wrong-site surgery and other Never Events across UK dental schools, highlighting institutional safety measures implemented in response to these events [13]. Aldahlawi et al. similarly identified patient-safety content within the undergraduate curriculum but demonstrated variation in students’ confidence across different safety competencies [14]. Halawany et al. found generally favourable self-reported patient-safety competence among dental students, including an understanding that reporting adverse events can promote change and reduce recurrence, although confidence varied across patient-safety domains [24]. Tufail et al. reported favourable perceptions of undergraduate patient-safety training and relatively high confidence in discussing and reporting errors, although responses varied when reporting was considered in relation to the severity or consequences of an error [20]. Adverse clinical events themselves were also used as learning opportunities. Ishikawa et al. examined 279 adverse events encountered during early clinical training and highlighted their potential value in helping students anticipate and understand potential clinical errors [21]. Collectively, these findings indicate that exposure to patient-safety concepts occurs during undergraduate dental education, but explicit and consistent preparation for recognizing reportable events and responding through appropriate reporting pathways remains variable across educational settings [14,20,24]. Although several studies evaluated students’ reporting-related knowledge, confidence, perceptions, or self-reported behaviours [19,20,24,26,27,29], formal assessment of reporting-specific competence was poorly described. The available evidence therefore provided limited information on whether students’ ability to recognize, document, and appropriately report clinical incidents was formally assessed within undergraduate dental education.

#### 3.3.2. Reporting Behaviour and Underreporting

Reporting behaviour varied considerably across the included studies, with evidence of a gap between awareness of patient safety and the consistent reporting of incidents. Tufail et al. reported generally favourable error-reporting confidence among dental students, although awareness varied across different patient-safety and error-related domains [20]. Padmanabhan et al. similarly identified variability in the documentation of potentially harmful events and near misses through formal incident or occurrence reporting processes [19]. Saraç Atagün and Çardakcı Bahar identified a marked discrepancy between exposure to errors and subsequent reporting; although 42.4% of participants reported having witnessed a medical error, only 3.4% reported an error, while awareness of the patient-safety notification system was also limited [27]. Underreporting was particularly evident in studies involving occupational clinical incidents. Zarabadipour et al. identified substantial underreporting of sharp-object injuries among dental students, with perceived insignificance of the injury reported as an important reason for non-reporting [26]. Antoniadou et al. also documented percutaneous injuries among undergraduate dental students within clinical training, highlighting the importance of structured reporting alongside preventive education [25]. Hassouneh et al. found that students were only marginally confident in raising patient-safety concerns, particularly when concerns involved the conduct of peers or clinical staff [29]. Taken together, the evidence suggests a gap between students’ awareness or confidence regarding patient safety and consistent reporting of incidents, particularly for incidents perceived as minor or in situations where students may be reluctant to raise concerns [19,20,26,27,29].

#### 3.3.3. Post-Event Review, Feedback, and Learning

A smaller group of studies described how incident and adverse-event reporting was linked to institutional review, feedback, and subsequent learning. Huertas et al. analyzed adverse events reported to the office of the clinical director in a dental school, providing direct evidence of how reported events can be captured and reviewed within an educational clinical setting [23]. Jain described an institutional initiative aimed at improving incident-reporting culture within a dental school clinic [28]. Osegueda-Espinosa et al. similarly emphasized the need for systematic collection and evaluation of adverse-event data arising during undergraduate clinical training [22]. Ishikawa et al. demonstrated another educational use of adverse events by incorporating students’ clinical incidents into structured learning activities, allowing events encountered during training to be reviewed and reflected upon [21]. Collectively, these studies suggest that the educational value of adverse events is enhanced when they are systematically reviewed and used to provide feedback, reflection, and opportunities for system-level learning [21,22,23,28].

#### 3.3.4. Barriers and Facilitators to Reporting

Barriers to incident and adverse-event reporting were identified at both individual and institutional levels. Hassouneh et al. reported that students’ willingness to raise patient-safety concerns was affected by fear of causing trouble, limited support, and concern that their reports might be ignored [29]. Tufail et al. showed that although students expressed confidence in reporting errors, responses were less consistent when the event did not result in patient harm, suggesting that perceptions of event severity may influence reporting decisions [20]. Zarabadipour et al. similarly identified perceived insignificance of an injury as an important reason for non-reporting of sharp-object injuries [26]. According to the studies, variability in awareness of reporting processes, institutional support, and opportunities for feedback also appeared to influence reporting-related behaviours [19,24,27]. In contrast, facilitators included clearly defined reporting pathways, supportive supervision, formal education on reporting, feedback following reported events, and a non-punitive institutional culture [19,28,29]. Overall, the findings indicate that students’ reporting behaviour is shaped not only by their knowledge and confidence, but also by the organizational environment in which reporting occurs.

## 4. Discussion

This scoping review mapped the available evidence on incident and adverse-event reporting within undergraduate dental education and identified a limited and heterogeneous evidence base. Across the 13 included studies, reporting-related content was commonly incorporated within broader patient-safety education rather than addressed as a distinct and consistently structured component of undergraduate curricula [14,24]. Although students demonstrated awareness of patient-safety principles and varying levels of confidence in reporting errors, this did not consistently translate into reporting behaviour [19,20,26,29]. Institutional factors also shaped reporting: the pathways available, the support students received from supervisors, whether feedback followed a report, and what students expected to happen if they raised a concern [19,26,28,29]. A smaller group of studies pointed to the educational potential of adverse events themselves, provided the events were reviewed afterwards and used to inform reflection, learning, or preventive action [21,22,23,28]. The principal gap, then, is not awareness of patient safety but the consistent integration of incident recognition, reporting, feedback, and learning into clinical education.

Education addressing incidents and adverse-event reporting specifically remains inconsistently represented within undergraduate dental curricula. Patient-safety principles were incorporated into formal teaching, but institutions differed in how far, and in what way, reporting-related competencies were covered [14]. The same variability appeared at the learner level, where students’ confidence and understanding differed across patient-safety risks, error reporting, and the discussion of errors [20,24]. That distinction matters: knowing about patient safety does not by itself equip a student to recognize a reportable event, judge when reporting is warranted or navigate the reporting pathway. Exposure to adverse clinical events may sharpen students’ recognition of potential errors [21], but experience alone is unlikely to prepare them consistently. Incident and adverse-event recognition and reporting therefore warrant explicit inclusion in undergraduate curricula, with defined learning outcomes and opportunities to build these competencies before and during clinical training [14,21].

Several studies examined students’ knowledge, confidence, perceptions, or self-reported behaviours related to patient safety and reporting [19,20,24,26,27,29]; however, there was limited evidence of formal assessment of students’ competence in recognizing and appropriately reporting clinical incidents. This suggests that although reporting-related concepts are addressed within broader patient-safety education, their assessment as a distinct educational competency remains insufficiently described.

The findings also indicated an apparent gap between students’ confidence or awareness regarding reporting and their actual reporting behaviour. Although dental students generally expressed willingness to report errors and discuss them with supervisors, confidence in reporting appeared less consistent when incidents were perceived as minor or had not resulted in patient harm [20]. This discrepancy was particularly evident for occupational incidents, with Zarabadipour et al. identifying substantial underreporting of sharp-object injuries and perceived insignificance of the injury as an important reason for non-reporting [26]. Related concerns were also evident in studies examining reported clinical injuries and students’ confidence and barriers in raising patient-safety concerns [25,29]. Underreporting is particularly relevant within dental education because failure to report an incident or near miss limits opportunities for identifying recurrent risks and implementing preventive measures. These findings suggest that developing reporting competence requires more than increasing students’ awareness of patient safety; students also need to understand the value of reporting events irrespective of their immediate clinical consequences and to regard reporting as an integral component of safe clinical practice [19,20,26,29].

The findings also highlight the importance of what occurs after an incident or adverse event is reported. Dargue et al. demonstrated the relevance of institutional responses to serious patient-safety incidents, reporting wrong-site surgery by students across UK dental schools and describing subsequent safety measures, including modified checklists, pre-operative briefings, and enhanced supervision [13]. Studies describing institutional reporting processes indicated that reported events could provide opportunities for systematic review, identification of contributing factors, and subsequent learning [23,28]. Huertas et al. demonstrated how adverse events reported within a dental school could be collated and analyzed, while Jain described an institutional initiative aimed at strengthening incident-reporting culture within a dental school clinic [23,28]. Similarly, adverse clinical events encountered by students have been used as a basis for reflection and learning during clinical education [21]. These findings suggest that reporting should not be viewed solely as a mechanism for documenting an event; its educational value is strengthened when reports are followed by review and meaningful feedback. Integrating this process into undergraduate clinical education may help students understand how individual incidents can contribute to wider system learning and risk reduction [23,28].

Several factors appeared to influence whether students translated their knowledge and intentions into reporting behaviour. Fear of causing trouble, inadequate support, and concern that reported issues might be ignored were identified as barriers to raising patient-safety concerns [29]. Perceptions regarding the significance of an event also influenced reporting, with minor or apparently inconsequential incidents being less likely to be reported [20,26]. At an institutional level, variability in awareness of reporting processes, opportunities for feedback, and institutional or supervisory support may further influence reporting-related behaviours [19,24,27]. Conversely, clearly defined reporting pathways, supportive supervision, formal education, and visible follow-up of reported events may facilitate reporting and reinforce its educational purpose [19,28,29]. These findings are particularly relevant in undergraduate clinical environments, where students work within supervisory and hierarchical structures and may be uncertain about the consequences of reporting. Creating a supportive and non-punitive reporting environment may therefore be as important as teaching students how to report, as reporting behaviour depends both on individual preparedness and on the institutional conditions in which reporting occurs [19,28,29].

The findings have practical implications for the design and delivery of undergraduate dental education. Incident and adverse-event reporting should be incorporated explicitly into clinical training rather than addressed only within broader patient-safety teaching [14]. Students should therefore receive explicit guidance on what constitutes a reportable incident or near miss, when and how such events should be reported, and the role of reporting within a patient-safety system [20,24]. Case-based discussion, structured reflection on adverse clinical events, and supervised review of incident reports all offer ways for students to build reporting competence before and during patient care [21,23]. These depend, however, on the clinic providing something to report into: accessible pathways within dental teaching clinics, feedback that arrives promptly, and a non-punitive stance that lets students report without fearing the consequences [19,28,29]. Linking reporting to the review and learning that follow it is what shifts the act from an administrative requirement to a meaningful part of clinical education and patient-safety improvement [21,22,23,28].

The findings are also consistent with broader approaches to patient-safety education. The WHO Patient Safety Curriculum Guide: Multi-professional Edition emphasizes learning from errors, understanding clinical risk, and familiarizing students with incident-reporting systems and processes within their training environments [6]. In dental education, Dargue et al. similarly reported increasing integration of formal patient-safety education and assessment across UK dental schools, although variation remained in the content and delivery of such education [30]. These broader educational perspectives support the explicit integration of reporting-related competencies within undergraduate dental curricula and reinforce the importance of linking incident reporting with subsequent learning and risk reduction [5,6,30].

This review has several strengths. The search was conducted across multiple bibliographic databases and supplemented by Google Scholar, with study selection guided by predefined eligibility criteria and reported in accordance with PRISMA-ScR [18]. The synthesis maintained a specific focus on incident and adverse-event reporting within undergraduate dental education, distinguishing reporting-related evidence from the broader literature on patient-safety culture. Nevertheless, several limitations should be considered. The available evidence was limited and heterogeneous in terms of study design, educational setting, participants, and reporting-related outcomes, which restricted direct comparison between studies. Much of the evidence was based on self-reported knowledge, confidence, attitudes, or behaviours rather than objectively measured reporting practices [19,20,24,27,29]. Some studies addressed reporting only as one component of broader patient-safety education or clinical-risk management [14,24], while others focused on specific events such as sharp or percutaneous injuries [25,26]. Because these events involve harm to the student rather than the patient, findings on their reporting may not generalize fully to patient-safety incidents, although both are managed through the same institutional reporting systems. The restriction to English-language literature, the capping of Google Scholar results at the first 100 records ranked by relevance together with the preliminary relevance filtering applied to those records before formal screening, and the absence of searches in additional education- or health-focused databases such as ERIC, CINAHL, or Embase may have resulted in relevant studies being missed. In addition, formal methodological quality or risk-of-bias appraisal was not undertaken, consistent with the evidence-mapping purpose of this review; consequently, the findings should be interpreted as a description of the available evidence rather than an assessment of its certainty or effectiveness. The review protocol was developed a priori but was not prospectively registered, for the reasons set out in Section 2.1.

The evidence gaps identified in this review highlight several priorities for future research. Incident and adverse-event reporting should be examined as a distinct competency within undergraduate dental education. Multicenter prospective studies are needed to establish how often incidents and near misses go unreported, and what drives that. Evaluations of educational interventions should reach past self-reported knowledge and confidence to capture what students actually report and how competently they do it. A third priority is the clinical environment itself: how institutional support, timely feedback, psychological safety, and freedom from punitive consequences shape reporting practice as students move through clinical training.

## 5. Conclusions

This scoping review mapped a small and heterogeneous body of evidence on incident and adverse-event reporting within undergraduate dental education. Across the thirteen included studies, reporting content was generally embedded within broader patient-safety teaching rather than addressed as a distinct competency, and few studies described how reporting knowledge or competence was formally assessed. Where reporting behaviour was examined, awareness and confidence did not consistently correspond to reported practice, although this observation rests largely on self-reported data drawn from a small number of single-institution studies. The barriers described most frequently related to uncertainty about what and how to report, concern about the consequences of reporting, and limited feedback once a report had been made, while the factors described as supportive centred on clear reporting pathways, accessible supervision, and a non-punitive institutional culture. Given the limited volume and heterogeneity of the available evidence, these findings should be regarded as an initial map of the field rather than as a basis for firm recommendations. They nonetheless suggest that incident and adverse-event reporting merits explicit curricular attention alongside the institutional conditions in which students are expected to report, and that future research should measure reporting behaviour directly rather than relying predominantly on self-reported knowledge and confidence.

## Figures and Tables

**Table 1:** Characteristics of included studies and their relevance to incident and adverse-event reporting in undergraduate dental education (n = 13).

Sl No.	Author (Year)	Country	Study Design	Participants/Setting	Relevance to Incident/Adverse-Event Reporting
1	Padmanabhan et al. (2025) [19]	UAE	Cross-sectional survey	Dental students, interns and clinical personnel in a dental teaching institution	Assessed incident/occurrence reporting of potentially harmful events, review of near misses, and perceptions related to a non-punitive reporting culture
2	Tufail et al. (2022) [20]	Pakistan	Multicenter cross-sectional study	200 third- and fourth-year dental students from four institutions	Assessed error-reporting confidence, including willingness to report one’s own and others’ errors, discuss errors with supervisors, and report errors that did not result in harm.
3	Ishikawa et al. (2024) [21]	USA	Retrospective educational study	Third-year predoctoral dental students; 279 adverse clinical events	Examined adverse clinical events encountered and presented by students and their use for reflection and learning during clinical education.
4	Osegueda-Espinosa et al. (2020) [22]	Mexico	Retrospective survey	207 dentists reporting experiences from undergraduate clinical training	Retrospectively examined adverse events experienced during undergraduate dental clinical training and highlighted the need for systematic collection and evaluation of adverse-event information.
5	Huertas et al. (2017) [23]	Colombia	Retrospective observational study	Undergraduate dental clinical setting	Analyzed adverse events reported to the Office of the Clinical Director, providing direct evidence on institutional adverse-event reporting within dental education.
6	Halawany et al. (2022) [24]	Saudi Arabia	Cross-sectional survey	490 undergraduate dental students and interns	Assessed patient-safety competence and students’ understanding of reporting adverse events, including the role of reporting in promoting change and reducing recurrence.
7	Dargue et al. (2021) [13]	United Kingdom	National cross-sectional survey	All 16 UK dental schools	Examined undergraduate dental schools’ experience of wrong-site surgery/Never Events, including reported student events and institutional patient-safety responses, providing evidence on adverse-event occurrence and reporting within undergraduate clinical education.
8	Aldahlawi et al. (2024) [14]	Saudi Arabia	Curriculum mapping and cross-sectional survey	Undergraduate dental curriculum, students and interns	Examined curricular coverage of clinical-risk management, including reporting of near misses and errors and incident monitoring, alongside learning from errors, and assessed student confidence in related patient-safety competencies.
9	Antoniadou et al. (2024) [25]	Greece	Retrospective observational study	Undergraduate clinical dental students	Examined reported sharps/percutaneous injuries among undergraduate dental students, including the circumstances of injury and immediate post-injury actions, within a structured prevention and reporting framework.
10	Zarabadipour et al. (2025) [26]	Iran	Quasi-experimental pre–post study	87 clinical dental students	Evaluated education concerning sharps injuries and identified substantial underreporting of incidents, including non-reporting when injuries were perceived as insignificant.
11	Saraç Atagün & Çardakcı Bahar (2025) [27]	Türkiye	Cross-sectional survey	323 dentistry and dental-assisting students	Assessed awareness of the patient-safety notification system, experience of witnessing errors, and error-reporting behaviour among dentistry and dental-assisting students.
12	Jain (2025) [28]	USA	Educational/quality-improvement study	Dental-school clinical setting involving senior dental students and faculty	Described an institutional initiative aimed at improving incident-reporting culture within a dental school clinical setting
13	Hassouneh et al. (2025) [29]	Jordan	National cross-sectional survey	Undergraduate dental students	Examined confidence, formal training, institutional support and barriers related to raising and reporting patient-safety concerns.

## Data Availability

The original contributions presented in this study are included in the article and Supplementary Materials. Further inquiries can be directed to the corresponding authors.

## Supplementary Materials

The following supporting information can be downloaded at: https://www.mdpi.com/article/10.3390/dj14100655/s1, Table S1: Database-specific search strategies used to identify studies on incident and adverse-event reporting in undergraduate dental education; File S1: Completed PRISMA-ScR checklist.

## Abbreviations

The following abbreviations are used in this manuscript:

**Abbreviation**	**Definition**
JBI	Joanna Briggs Institute
PCC	Population–Concept–Context
PRISMA-ScR	Preferred Reporting Items for Systematic Reviews and Meta-Analyses extension for Scoping Reviews
WHO	World Health Organization
