# Supplementary material for: Incident and Adverse-Event Reporting in Undergraduate Dental Education: A Scoping Review of Educational Evidence, Curricular Integration, Perceptions, and Barriers
Source: Dent J (Basel). 2026 Oct 9;14(10):655. doi: 10.3390/dj14100655 (PMC13703497; doi:10.3390/dj14100655)
Supplement: Supplementary file 1 [file dentistry-14-00655-s001.zip › Table S1.pdf]

Supplementary Table S1. Database-specific search strategies used to identify studies on incident and adverse-event reporting in undergraduate dental education

Database	Search strategy	Records entered into reference management/screening (n)
**PubMed**	("incident reporting" OR "adverse event*" OR "clinical incident*" OR "patient safety" OR "near miss*" OR "error reporting") AND ("dental student*" OR "undergraduate dental education" OR "dental education" OR "dental school*" OR "predoctoral dental") AND ("education" OR "curriculum" OR "training" OR "teaching" OR "learning" OR "competence*" OR "assessment")	**175**
**Scopus**	TITLE-ABS-KEY ("incident reporting" OR "adverse event*" OR "clinical incident*" OR "patient safety" OR "near miss*" OR "error reporting") AND TITLE-ABS-KEY ("dental student*" OR "undergraduate dental education" OR "dental education" OR "dental school*" OR "predoctoral dental") AND TITLE-ABS-KEY (education OR curriculum OR training OR teaching OR learning OR competence* OR assessment)	**108**
**Web of Science**	*TS=("incident reporting" OR "adverse event" OR "clinical incident*" OR "patient safety" OR "near miss*" OR "error reporting") AND ("dental student*" OR "undergraduate dental education" OR "dental education" OR "dental school*" OR "predoctoral	**107**

	dental") AND (education OR curriculum OR training OR teaching OR learning OR competence* OR assessment)	
**Google Scholar**	("incident reporting" OR "adverse event" OR "clinical incident" OR "patient safety" OR "near miss" OR "error reporting") AND ("dental student" OR "undergraduate dental education" OR "dental education" OR "dental school" OR "predoctoral dental") AND (education OR curriculum OR training OR teaching OR learning OR competence OR assessment)	**27***
**Total**		**417**

Search date: May 20, 2026.

Limits: English-language studies published between 2015 and 2026.

*For Google Scholar, the first 100 results ranked by relevance were reviewed. Of these, 27 potentially relevant records were selected for export to EndNote and subsequent screening and therefore contributed to the 417 records identified.

Additional searching: Reference lists of included studies were manually screened, and forward citation searching was undertaken using Google Scholar to identify additional potentially eligible studies. No additional studies meeting the eligibility criteria were identified through these searches.

Abbreviations: TITLE-ABS-KEY, title, abstract, and keywords.Top of Form
